# Digital DNA-DNA hybridization for microbial species delineation by means of genome-to-genome sequence comparison

**DOI:** 10.4056/sigs.531120

**Published:** 2010-01-28

**Authors:** Alexander F. Auch, Mathias von Jan, Hans-Peter Klenk, Markus Göker

**Affiliations:** 1 Center for Bioinformatics Tübingen, Eberhard-Karls-Universität, Tübingen, Germany; 2 DSMZ – German Collection of Microorganisms and Cell Cultures GmbH, Braunschweig, Germany

**Keywords:** *Archaea*, *Bacteria*, BLAST, GBDP, genomics, MUMmer, phylogeny, species concept, taxonomy

## Abstract

The *pragmatic* species concept for *Bacteria* and *Archaea* is ultimately based on DNA-DNA hybridization (DDH). While enabling the taxonomist, in principle, to obtain an estimate of the overall similarity between the genomes of two strains, this technique is tedious and error-prone and cannot be used to incrementally build up a comparative database. Recent technological progress in the area of genome sequencing calls for bioinformatics methods to replace the wet-lab DDH by *in-silico* genome-to-genome comparison. Here we investigate state-of-the-art methods for inferring whole-genome distances in their ability to mimic DDH. Algorithms to efficiently determine high-scoring segment pairs or maximally unique matches perform well as a basis of inferring intergenomic distances. The examined distance functions, which are able to cope with heavily reduced genomes and repetitive sequence regions, outperform previously described ones regarding the correlation with and error ratios in emulating DDH. Simulation of incompletely sequenced genomes indicates that some distance formulas are very robust against missing fractions of genomic information. Digitally derived genome-to-genome distances show a better correlation with 16S rRNA gene sequence distances than DDH values. The future perspectives of genome-informed taxonomy are discussed, and the investigated methods are made available as a web service for genome-based species delineation.

## Introduction

Macroscopic organisms, such as animals, plants and fungi, are generally easy to distinguish for species classification by an abundance of morphological differences, behavioral traits, or by interbreeding barriers. For microorganisms belonging to the two ‘prokaryotic’ domains of life,  *Archaea* and *Bacteria* [1], species delineation is a much more challenging task. Morphological features and metabolic peculiarities can be used to classify microorganisms to a certain degree of confidence, but the number of features and peculiarities that can easily be recognized for differentiation is rather limited. Consideration of genetic – and nowadays increasingly genomic – features often enables a deeper resolution for the differentiation, placing DNA-DNA hybridizations (DDH) in a key position as a major tool in microbial species delineation [2-4]. Starting in the early 1970s [5-7], several methods to determine DDH values have been developed [8]. The general principle of DNA-DNA re-association requires (i) shearing the gDNA of the assayed organism and the gDNA of the reference organism(s) (type strain(s)) into small fragments of 600-800 bp; (ii) heating the mixture of DNA fragments from both strains to dissociate the DNA double-strands; and (iii) subsequently decreasing the temperature until the fragments reanneal. For the reason that the melting temperature of a double-strand depends on the degree of matching base pairings between both strands, genomic (dis-)similarity can be inferred from the melting temperature. The hybrid DDH value is usually specified relative to the DDH value obtained by hybridizing a reference genome with itself. DDH values ≤70% are considered as an indication that the tested organism belongs to a different species than the type strain(s) used as reference(s) [2,4].

All established variations of DDH determination are technically demanding, labor-intensive and time-consuming procedures, therefore DDH determination is now performed by only a few specialized laboratories, and microbial taxonomists apply DDH only in cases where the strains to be differentiated have previously been shown to be closely related in terms of their 16S rRNA gene sequences [3,4]. In practice, the distinct DDH determination methods are all based on the same principle, but frequently lead to different results [8,9]. Accordingly, there is increasing interest to replace DDH with more reproducible and absolute methods that do not require the repeated use of reference strains over and over again. Enabled by the automation of Sanger sequencing (in the 1990s) and the now dominating pyrosequencing methods [10], the rapid technical progress in sequencing technology lets us envision that genome sequencing will very soon become a routine analytical method for microbial species delineation. This situation resembles events in the mid 1980s when the traditional DNA:rRNA hybridization [11] was first technically improved [12] but then rapidly and completely replaced by the use of 16S rRNA sequences [1], which could be stored in databases that (apart from resequencing to resolve artifacts and 16S rRNA heterogeneity) require only one experiment (sequence) per type strain to fix it forever in a rapidly growing and seemingly unlimited database [13]. The availability of whole genome sequences has dramatically changed the way microbiologists formulate and answer questions about subjects of interest, often termed the'-omics revolution' [14]; the time has now arrived to use genome sequences in the daily routine of microbial taxonomists for the purpose of species delineation.

Some *in silico* methods based on the comparison of completely sequenced genomes have already been suggested as an alternative to DDH [15,16]. Goris *et al.* [17] applied BLAST [18] to determine high-scoring segment pairs (HSPs) between genome sequences after cutting them into small 1000 bp-long pieces to emulate the DDH procedure (see above). The 'Average Nucleotide Identity' (ANI) and the 'Percentage Conserved DNA' were then calculated from the sets of HSPs. The method was implemented in a Perl script that could be obtained from its authors on request. Regression analyses of the data suggested that the resulting *in-silico* genomic similarity measures were in good agreement with DDH values determined for the same pairs of strains in the wet lab [17].

Here we try to expand this sequence-based approach. A variety of similarity search methods have been established in addition to BLAST for the analysis of HSPs [19,20], along with a number of algorithms to calculate genome-to-genome distances (GGD) that can be used to infer phylogenies [21-25]. What remains to be established is whether these methods/algorithms could turn out to be more suitable to mimic DDH *in silico*. For instance, experience has been gained on how to adapt GGD approaches such as genome BLAST distance phylogenies (GBDP) to conditions such as large numbers of genomic repeats and heavily reduced genomes [21,24]. GGD methods have already been shown to be rather valuable tools in reconstructing whole-genome based trees of *Archaea* and *Bacteria* [25]. Therefore it would be very interesting to see if the same methods could also be used to estimate species boundaries, much like the 16S rRNA gene sequences are used for both inferring phylogenies and calculating pairwise dissimilarities between strains, in order to assess whether they need to be subjected to DDH for drawing conclusions about their species status [4].

In the present study, we compare the major state-of-the-art programs for determining high-scoring segment pairs (HSPs) and maximally unique matches (MUMs) [20], as well as previously described approaches for calculating GGD from such sets of HSPs or MUMs, regarding their performance in an *in-silico* framework to replace DDH in comparison to ANI [17]. We also aim at enlarging the empirical set of data and at improving the statistics used for assessing the performance of such methods. As a further important selection criterion for GGD approaches, we also examine their relative computational running times and memory requirements. Correlation between 16S rRNA and DDH data is of practical interest because 16S sequencing is a less tedious and error-prone task than DDH, and sufficiently high 16S distances can predict DDH similarities below 70%  [26]. Moreover, while 16S rRNA gene sequences are themselves limited in estimating evolutionary distances (after all they represent only about 0.1% of the coding part of microbial genomes), it can nevertheless be used according to the *ceteris paribus* principle to assess the precision of either GGD or DDH. We thus were interested in the correlation of either method with 16S rRNA distances. Finally, we investigate the performance of GGD on artificially incomplete genomes. This is of considerable practical relevance, because gap closure in draft genome sequences is a very time-consuming process involving primer walking to create finishing reads, as well as frequent rounds of re-assemblies [27,28]. Therefore, it is of interest to elaborate which minimal fraction of a genome sequence might be required for a reliable estimation of GGD.

This work is the basis for an accompanying standard operating procedure for conducting HSP- or MUM-based genomic comparisons [29] and for a web service that implements this procedure (http://ggdc.gbdp.org/).

## Material and Methods

### Empirical data

The first part of the dataset used in the empirical tests, i.e. pairs of completely sequenced genomes and corresponding DDH values, is the one used in [17], which comprises distinct 'hybridization groups', i.e. sets of strains from one or few genera that have been compared to each other. Additional data were obtained by (i) determining a set of type strains for which whole genomes are available. This was done by reconciling the Genomes On Line database [30] and the DSMZ database (http://www.dsmz.de/microorganisms/); and by (ii) screening the *International Journal of Systematic and Evolutionary Microbiology* (http://ijs.sgmjournals.org/) for articles containing DDH values of these strains. Consequently, because the size of the dataset could be increased by 50%, the final list of genomes and DDH values comprised 93 genome/DDH pairs. Some of the additional genomes were not completely sequenced at the time of downloading but comprised distinct contigs from shotgun sequencing. These details are included in the full genomes list contained in the Electronic Supplementary Material (ESM). Unfortunately, DDH information is usually only available for type strains, whose genomes comprise only a minor proportion of the currently available fully sequenced microbial genomes [31,32].

### Determining HSPs and MUMs

The software packages used for determining HSPs were NCBI-BLAST version 2.2.18, WU-BLAST version 2.0MP-WashU (04-May-2006) [18], BLAT version 34 [19] and BLASTZ version 7 [33]; MUMs were determined with MUMmer version 3.0 [20]. In the case of BLASTZ, we additionally investigated alternative settings of the “K” parameter (2000, 2500, 3000, 3500); this parameter determines the minimum raw score required for a HSP for further consideration. In the case of BLAT, we used either 0%, 50%, or 90% (default) as minimum sequence identity required within HSPs and 0 (for 0%) or 30 (for 50% and 90% minimum identity) as corresponding minimum scores. Lowering these values was expected to provide more accurate results. For the most sensitive setting, we additionally lowered the tile size from 12 (default) to 8; the tile size approximately behaves like the word length parameter of the BLAST programs. Regarding the settings of MUMmer, we applied minimum match lengths ranging between 16 and 50 and the three possible settings for the treatment of matches in both forward and reverse strand (command-line switches -mum, -mumreference and -maxmatch). The modified command-line switches are also shown in Table 1. In either case, the resulting data were stored in CGVIZ format [34] for further proceeding with GBDP as described below. For MUMmer, we used the MUM length as a replacement for the HSP score, which is, of course, not available from that program.

**Table 1:** Parameters, computation times and memory consumption of programs applied to selected genome pairs.

**Program**	**Parameters (and** **abbreviation)**	**Running time (user ** **and system) in seconds**	**Memory consumption** **in Mb**
**HSP/MUM determination**			
NCBI-BLASTN	(ncbiblastn)	25.79	165.92
WU-BLASTN	(wublastn)	896.79	904.69
BLASTZ	K=2000 (blastz2000)	48.11	125.73
	K=2500 (blastz2500)	47.80	124.39
	K=3000 (blastz3000)	47.64	123.19
	K=3500 (blastz3500)	47.61	122.45
BLAT	-minScore=0 – minIdentity=0 – tileSize=8 (blatminTS)	2,134.54	2443.43
	-minScore=0 – minIdentity=0 (blatmin)	178.15	174.78
	-minScore=30 – minIdentity=50 (BLAT)	177.05	167.41
	-minScore=30 – minIdentity=90 (blatid90)	177.08	167.41
MUMmer	-l 20 -mum (mum20)	13.74	94.84
	-l 30 -mum (mum30)	13.76	94.52
	-l 40 -mum (mum40)	13.77	94.31
	-l 50 -mum (mum50)	13.72	94.17
	-l 20 -mumreference (ref20)	13.80	93.44
	-l 30 -mumreference (ref30)	13.81	93.44
	-l 40 -mumreference (ref40)	13.79	93.44
	-l 50 -mumreference (ref50)	13.75	93.44
	-l 20 -maxmatch (max20)	14.07	93.44
	-l 30 -maxmatch (max30)	13.95	93.44
	-l 40 -maxmatch (max40)	13.86	93.44
	-l 50 -maxmatch (max50)	13.88	93.44
**Distance calculation** **with GBDP** **(all formulas in each case)**			
tr F-2	mean, all programs	6,169.98	1836.62
cov F-2		22.22	424.68
tr NF		6,178.77	1853.68
cov NF		22.51	404.98
tr F-2	mean, all programs except MUMmer	39.54	415.04
cov F-2		12.18	229.58
tr NF		111.19	506.07
cov NF		19.78	235.64
tr F-2	mean, NCBI-BLASTN only	155.83	732.63
cov F-2		34.89	465.39
tr NF		206.68	942.36
cov NF		43.13	473.68
tr F-2	mean, MUMmer only	11,278.68	3021.26
cov F-2		30.60	587.26
tr NF		11,235.07	2976.68
cov NF		24.78	546.09

**Table 1****.** Performance of HSP/MUM determination programs under distinct settings (parameters are given if deviating from the default) and the subsequent distance calculation with GBDP. Abbreviations: cov, coverage distances; tr, greedy-with-trimming distances; F-2, filtering using an e-value threshold of 10^-2^; NF, no filtering.

### Distance calculation

Pairwise distances between genomes were calculated with GBDP. All programs determining HSPs were run with or without HSP filtering, i.e. removing all HSPs with an e-value larger than 10^-2^ prior to calculating distances. The next step is to remove overlapping parts of HSPs in either genome using the so-called greedy-with-trimming algorithm [24]. This procedure proved to be valuable in phylogenetic inference from genomes with large numbers of repeats, but trimming may also be omitted (the resulting distance formula being called 'coverage distance') [24]. Finally, distances are calculated from the sets of (remaining) HSPs using one of several approaches.

Let *H_xy_* denote the total length of all HSPs and *I_xy_* denote the sum of the number of identical base pairs over all HSPs found by BLASTing genome *x* against genome *y*, whereas *H_yx_* and *I_yx_* are obtained by using *y* as the query and *x* as the subject sequence. GGD can then be defined as follows [21,24,35]:


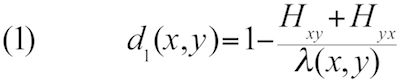



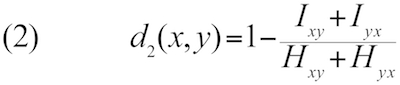



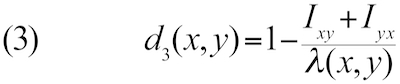


Here λ(x,y) is a function of the lengths of the two genomes; in the simplest case, lambda is equal to the sum of the genome lengths.

Other distance functions can be derived from the previously mentioned ones by applying a logarithmic transformation or by using twice the length of the shorter genome instead of the sum of the genome lengths, resulting in a total number of ten distance functions to be tested. (The internally applied numbering of the GBDP software is: 0-3: formula (1); 4-5: formula (2); 6-9: formula (3); 0, 2, 6, 8: sum of genome lengths in denominator; 1, 3, 7, 9: twice the minimum genome length in denominator; 0, 1, 4, 6, 7: no logarithm; 2, 3, 5, 8, 9: logarithm. Further details are provided in the ESM.) Using the minimum genome length improved phylogenetic accuracy if heavily reduced genomes were considered [21,24]; logarithmic transformation of the data was also useful in such cases, but had no effect on the non-parametric correlation analyses in the present study (see below; one of the advantages of our approach). Note that we here examine methods based on the comparisons of the underlying nucleotide sequence only; while distance approaches using the translated amino acids may perform better in phylogenetic inference of deep nodes [21], DDH is concerned with closely related organisms only, and mimicking it *in silico* based on direct genome sequence comparisons is straightforward.

### Quantifying method performance

Goris *et al* [17] used linear regression to determine the suitability of their ANI algorithm to mimic DDH values. This regression procedure has two disadvantages. First, it presupposes a linear relationship between DDH values and genome distances, which may or may not hold for current GGD approaches. For instance, some GGD formulas result in distances more rapidly saturated than others, that is, distances that do not show significant additional increase in spite of further increasing genomic differences [21,24]. This problem can be overcome by replacing regression with correlation and Pearson's with Kendall's non-parametric correlation coefficient [36,37] which uses the values' ranks only (in the following, Pearson's coefficients are included for selected values for comparison; full results for either coefficient are available in the ESM). Moreover, non-parametric statistics are more robust against outliers. In contrast to Goris *et al.* [17], we calculate distance functions; that is, a correlation of -1.0 is optimal. As in the following, all correlations, as well as all plots (see below), were computed with the R package [38].

Secondly, many users will only be interested in the error ratio of the whole-genome distance regarding the question whether the DDH value is lower than 70%. This problem can be solved by applying a two-step procedure: (i) determining, for each GGD approach, the distance threshold *T* resulting in the smallest error ratio, and (ii) reporting this optimal error ratio. Here, error ratio is defined as the sum of the number of false positives (distances at most as large as *T* corresponding to DDH values lower than 70%) and false negatives (distances larger than *T* corresponding to DDH values at least as large as 70%) divided by the total number of pairwise distances. The optimal *T* can then be used in real genome comparisons for replacing the DDH approach, too. We estimated the optimal *T* by assessing all values between the maximum and the minimum for each GGD variant, applying a step width of 1/1000 of the range.

To compare the results obtained with GBDP to those obtained with the ANI and 'Percentage Conserved DNA' methods as reported by Goris *et al.* [17], we reduced the dataset to the genome pairs examined in the latter study. We could not apply ANI to the full dataset, because when applying the ANI Perl script of Konstantinidis to the genome pairs analyzed in the latter study, we could not adequately corroborate the results reported in [17]. For the sake of convenience we used the results for the 62 genome pairs analyzed with ANI directly as published in [17] to compare the performance of ANI with the GGD methods assessed in the present study. Deloger *et al* [16] apparently reimplemented the ANI method but did not disclose whether they obtained the same results as in [17] when applied to the same strains.

In order to correlate GGD and DDH with pairwise distances inferred from the 16S rRNA, this gene was extracted from all completed and annotated genomes under study, resulting in a set of 59 pairs of genomes. The 16S rRNA gene sequences were aligned with Poa v2 in progressive alignment mode [39], and uncorrected (“p”) distances were calculated from the aligned sequences using PAUP* v4b10 [40] under the MISSDIST=IGNORE setting. Correlations were calculated as described above.

### Run-time and memory consumption measurements

Computation time of the programs determining HSPs or MUMs as well as of GBDP applied to these data was measured using a reduced dataset comprising a selection of eight Genomes of the *Escherichia*/*Shigella* group. Plasmids were removed from the dataset, only chromosomal data was used. On the one hand, this allowed us to compare job running times, since all FASTA files had approximately the same size (5 Mbp). On the other hand, using closely related strains leads to the detection of a considerable amount of HSPs by the different local alignment search tools, thus allowing estimates of an upper bound for the search time. These measurements were performed on a AMD Quad-Core Opteron System equipped with a 2.3 GHz CPU and 20 GB RAM.

### Simulation of incomplete genome sequencing

In order to measure method performance on incompletely sequenced genomes, artificial gaps were incorporated into the fully sequenced genomes of the empirical datasets (that is, 62 pairs of complete genomes formed the basis of our simulation). This was based on the well-known Lander-Waterman formula [41], which is usually applied to estimate the sequencing effort necessary to obtain a given coverage, as follows. Based on a realistic value of 700 bp as the fixed read length (http://www.jgi.doe.gov/sequencing/statistics.html), the real length of the fully sequenced chromosome or plasmid, and the proportion of the genome to be retained, the number of reads necessary to achieve this proportion is calculated using the Lander-Waterman approach [41]. An array of all positions in the original genome is created, and all positions are marked as 'not sequenced'. A starting position in the genome for each of the calculated number of reads is then drawn at random, and this one as well as the corresponding 699 downstream array positions are marked as 'sequenced'. After all reads have been considered, positions remaining in 'not sequenced' state are then removed from the input genome, creating disjoint contigs, which are output. Applied several times, this procedure creates modifications of input genomes whose lengths are dispersed around an expected value equal to the original genome length times the input sequencing proportion.

Based on this algorithm, a total number of 100 simulation runs was conducted for sequencing proportions of 0.99, 0.95, 0.90, 0.85, 0.80, 0.75, 0.70, 0.60, 0.50, 0.40, 0.30, 0.20, and 0.10. Note that this approach corresponds to simulating genomes that are incompletely sequenced, but are nevertheless lacking sequencing errors in all reads and are correctly assembled. A simulation including an assembly of artificially created reads in each replicate has been rejected for reasons of running time. On account of this, only a single, reasonably fast and well-performing HSP determination approach was examined in simulation. Method performance on incomplete genomes was quantified in two ways: First, error ratios were determined after applying the optimal threshold as determined for the corresponding distance function and complete genomes (see above). Second, Euclidean distances between the GGD calculated from the original genomes and the GGD inferred from the respective incomplete genomes were calculated using the Eukdis program [42].

## Results

### Correlation of GGD algorithms with and prediction of DDH values

**Figure 1:**
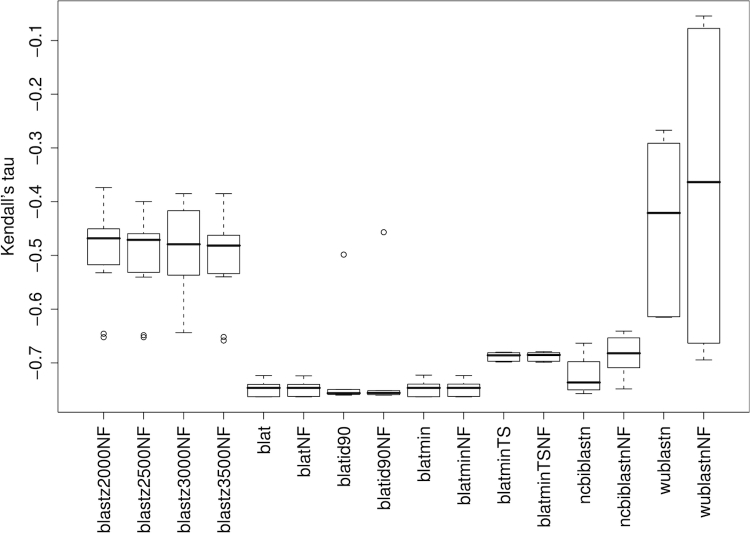
Non-parametric correlations of DDH values with distances based on HSP determination. Each boxplot comprises Kendall's correlation coefficients calculated for all GBDP distance formulas and the selected program for HSP determination. Note that lower values indicate better DDH prediction. For abbreviations of the method-parameter combinations, see Table 1; 'NF' was added if HSP filtering was not applied.

Results from non-parametric correlation of DDH values with distances based on HSP determination programs are shown in Figure 1. The best performing software was BLAT, followed by NCBI-BLASTN; BLASTZ and particularly WU-BLASTN performed less well. The globally best Kendall correlation obtained was -0.763 from the combination of BLAT under default values with or without HSP filtering ('blat' and 'blatNF' in Fig. 1), trimming and either formula (3) or its logarithmic modification. The overall performance of BLAT could not be increased further by setting the minimum sequence identity required within HSPs and the corresponding minimum scores to their minimal possible value ('blatmin' and 'blatminNF'); combined with this setting, also formula (3) and its logarithmic modification performed best, also achieving a correlation of -0.763. In contrast, requesting a minimum within-HSP identity of 90% ('blatid90'/'blatid90NF', best correlation -0.760) and particularly setting the tile size to its minimum value of 8 ('blatminTS'/'blatidminTSNF', best correlation -0.699) decreased the performance. NCBI-BLASTN optimally achieved a correlation of -0.757 (same distance functions, filtering), WU-BLASTN one of -0.695 (same distance functions, no filtering), BLASTZ one of -0.658 (K set to 3500, no filtering, distance formula (2)). In either case, trimming outperformed coverage-based distances. Regarding Pearson's r, BLAT showed the most pronounced correlation of -0.954 (when combined with 90% minimum identity within HSPs, trimming and formula (3)), followed by NCBI-BLASTN (-0.925; no filtering, trimming, formula (3)).

**Figure 2:**
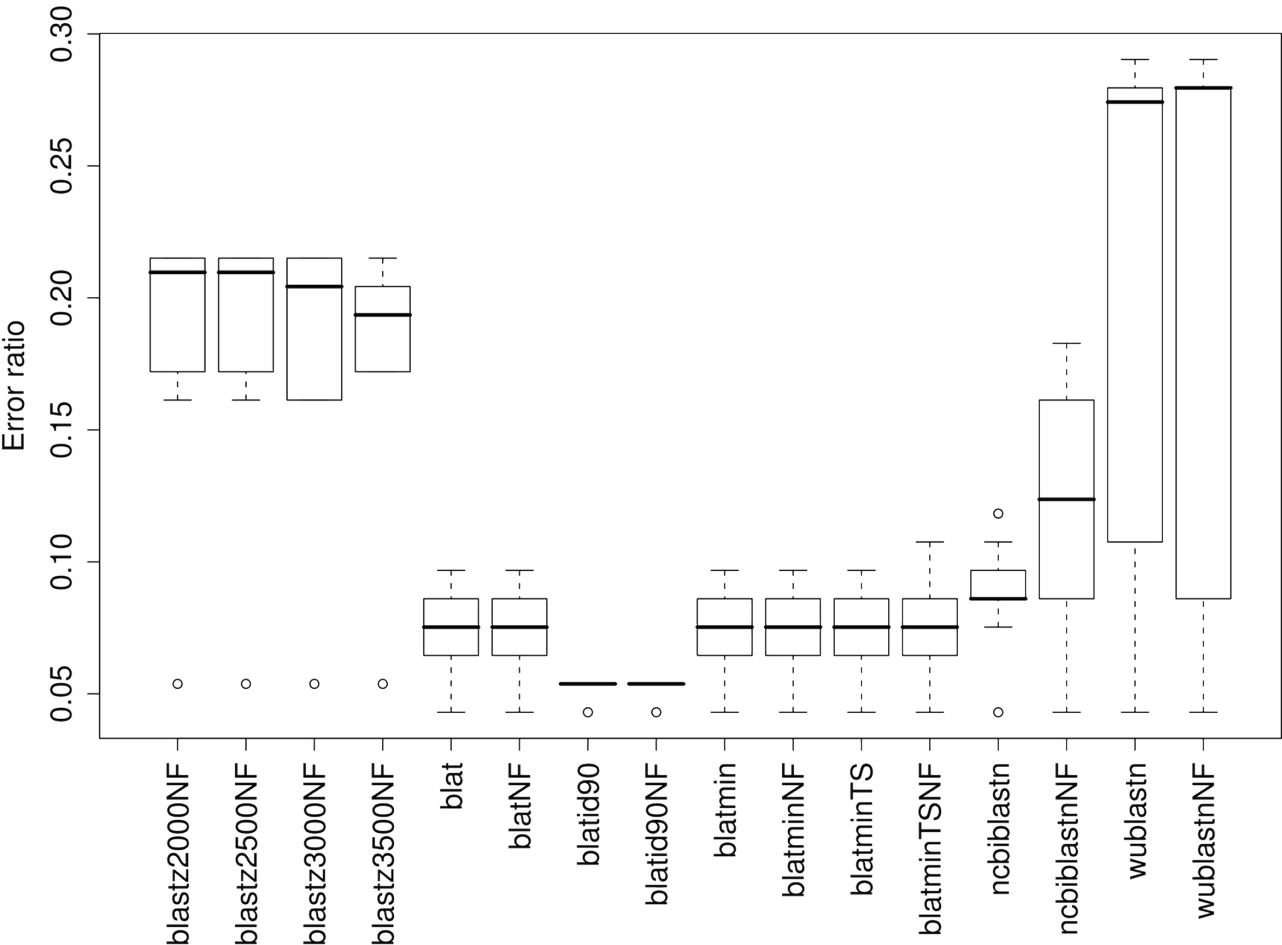
Error ratios of distances based on HSP determination in predicting whether DDH values are at least as large as 70% or lower. Each boxplot comprises error ratios calculated for all GBDP distance formulas and the selected program for HSP determination. For abbreviations of the method-parameter combinations, see Table 1; 'NF' was added if HSP filtering was not applied.

In terms of the error ratio (Figure 2), a combination with at least one GBDP distance function that resulted in the globally lowest value of 0.043 was present for all programs except BLASTZ (lowest ratio 0.054). However, median error ratios over all distance functions largely mirrored the relationships between the correlations. The settings resulting in the best correlation for each program also resulted in minimal error ratios, but not necessarily with the same distance functions. In contrast, here formula (2) and its logarithmic modification were optimal for all HSP determination programs. The best GGD threshold was 0.044 and 0.045, respectively, for NCBI-BLASTN without filtering, and 0.042 and 0.043, respectively, for the best BLAT-based variant.

**Figure 3:**
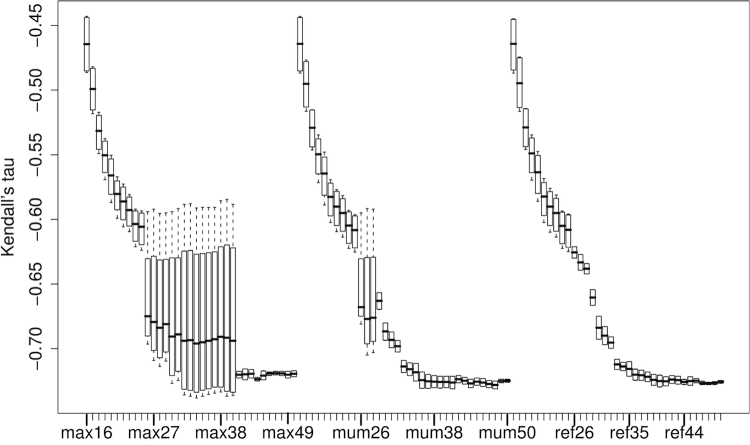
Non-parametric correlations of DDH values with distances based on MUMmer. Each boxplot comprises Kendall's correlation coefficients calculated for all GBDP distance formulas, the greedy-with-trimming algorithm and the selected MUMmer parameter combination. Note that lower values indicate better DDH prediction. The x-axis comprises the three investigated series of minimum MUM lengths ranging between 16 and 50, one series per setting for the treatment of matches in both forward and reverse strand, abbreviated max, mum and ref, respectively. For the meaning of these abbreviations, see Table 1.

**Figure 4:**
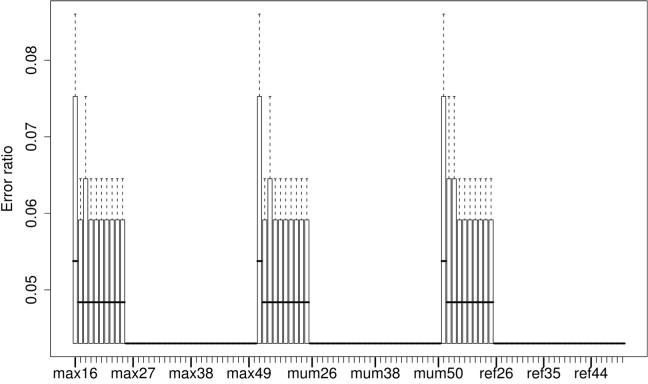
Error ratios of distances based on MUMmer in predicting whether DDH values are at least as large as 70% or lower. Each boxplot comprises error ratios calculated for all GBDP distance formulas and the selected MUMmer parameter combination. The x-axis comprises the three investigated series of minimum MUM lengths ranging between 16 and 50, one series per setting for the treatment of matches in both forward and reverse strand, abbreviated max, mum and ref, respectively. For the meaning of these abbreviations, see Table 1.

Results from non-parametric correlation of DDH values with distances based on MUMmer are shown in Figure 3. The best Kendall correlation value of -0.749 was obtained with a minimum MUM length of 44 bp in combination with -mum, the GBDP variant coverage instead of trimming, and formula (1) and its logarithmic modification. The best Pearson correlation with DDH (-0.935) was obtained with the same settings but -maxmatch instead of -mum. In contrast, the minimal error ratio of again 0.043 was obtained by all methods for sufficiently large minimum MUM lengths (Figure 4).

**Figure 5:**
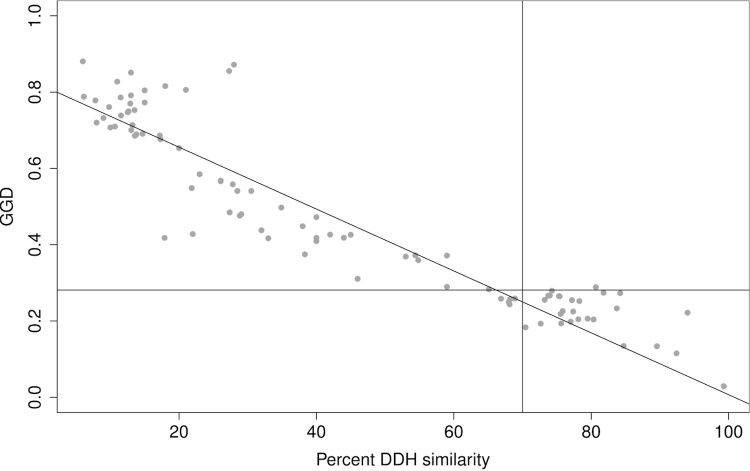
Scatterplot of DDH (x-axis) and GGD inferred with BLAT under default values without HSP filtering, greedy-with-trimming and formula (3). The vertical line indicates the 70% DDH threshold, the horizontal line indicates the GGD threshold that results in the lowest error ratio for these settings. The result of a robust-line fit using the R function *line(*) is also shown, indicating that regression and determination of a threshold with lowest error ratio may differ in their estimates of a GGD threshold to replace the DDH 70% cutoff.

**Figure 6:**
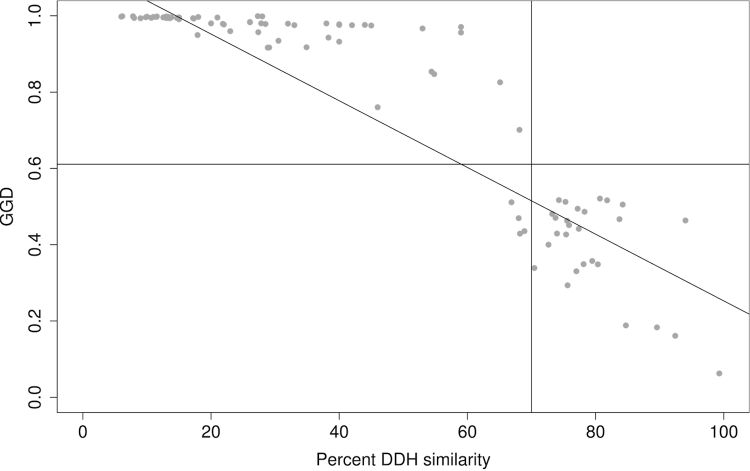
Scatterplot of DDH (x-axis) and GGD inferred with MUMmer using a minimal MUM length of 44 bp, no trimming and formula (1). The vertical line indicates the 70% DDH threshold, the horizontal line indicates the GGD threshold that results in the lowest error ratio. For the robust-line fit, see caption of Figure 5. The plot shows that MUMmer-based GGD more rapidly reaches saturation than HSP-based GGD and that it is not linearly related to DDH, underlining the need to rely on rank-based correlation coefficients for an unbiased comparison of distance functions.

Scatterplots of DDH and HSP-based and MUM-based GGD (under the best settings for each) are shown in Figure 5 and Figure 6; relevant DDH and GGD thresholds are indicated.

### Comparison of GGD with ANI and 16S rRNA

In the reduced dataset of 62 genome pairs for which ANI and 'Percentage Conserved DNA' values were available [17], NCBI-BLASTN combined with greedy-with-trimming, no filtering and formula (3) or its logarithmic version as distance functions performed best and achieved a non-parametric (Kendall) correlation of -0.771. The best BLAT-based method (correlation: -0.760) was also the same as above for the full dataset. In contrast, correlation of DDH with ANI was 0.717 and of DDH with 'Percentage Conserved DNA' was 0.686 (full results are provided in the ESM). Regarding Pearson's correlation coefficients, relative performance of ANI (0.974) and 'Percentage Conserved DNA' (0.977) was higher; they were outperformed by BLAT combined with greedy-with-trimming and formula (3) only (0.979; for details see ESM). Note that ANI and 'Percentage Conserved DNA' are similarity methods and, hence, the sign of the correlations must be inverted to compare them with GGD. The respective minimal error ratios: ANI, 0.065; 'Percentage Conserved DNA', 0.081; NCBI-BLASTN, 0.065; BLAT, 0.065. Thus, the best GGD approaches analyzed in the present study slightly outperform the previously described methods [17] if applied to the same dataset.

The combination of BLAT, formula (1), trimming and 90% minimum identity within HSPs showed the highest correlation (0.612 as Kendall coefficient) with 16S rRNA distances; for WU-BLASTN, a maximum of 0.535 (settings were: formula (3), trimming and no filtering), for NCBI-BLASTN, 0.602 (formula (1), trimming, no filtering), for BLASTZ, 0.503 (formula (1), K set to 3500, no trimming, no filtering), and for MUMmer, 0.611 (-mumreference, 44 as minimum MUM length), was achieved. In terms of the Pearson coefficient, BLAT also performed best (0.896; formula (1) with or without trimming and filtering). Interestingly, DDH displayed a much less pronounced correlation with 16S rRNA distances than the GGD approaches closest to the 16S (Kendall's tau: -0.533; Pearson's r: -0.745; note that the sign is reversed because distances and similarities were correlated).

In the further reduced dataset (55 genome pairs) comprising the genome pairs for which ANI values were also available, MUMmer (0.616; -maxmatch, 44 as minimum MUM length) displayed the highest correlation with 16S distances, as well as BLAT and NCBI-BLASTN (0.616, settings as in the last paragraph). ANI and 'Percentage conserved DNA' obtained a slightly more pronounced Kendall coefficient of -0.618 and -0.621, respectively. In terms of Pearson's r, BLAT performed best (0.899; formula (1), trimming, 90% minimum identity within HSPs), followed by NCBI-BLASTN (0.890; no filtering, trimming, formula (1)) whereas ANI (-0.832) and particularly 'Percentage conserved DNA' (-0.742) correlated less well. DDH performed approximately as above (-0.561/-0.774).

### Running time and memory consumption

The computation times of the distinct programs if applied to selected genome pairs are shown in Table 1. MUMmer performs best regarding its needs for both time and space; the parameters regulating minimum match length and treatment of matches in the two strands have negligible impact. However, the fastest HSP determining software, NCBI-BLASTN, is only about half the speed as MUMmer, even though the BLAST algorithm is considerably more complex. BLASTZ again is approximately half the speed than NCBI-BLASTN, followed by BLAT. While the performance of BLASTZ is hardly affected by modifying the settings, BLAT memory consumptions dramatically increases with lower tile size. By far the worst performing program regarding both time and space is WU-BLASTN.

CPU time and memory consumption during the distance calculation with GBDP generally benefits from using the simpler coverage functions. This effect is particularly prominent in the case of MUMmer, most likely because the MUMs created by this program are more numerous and shorter than the HSP output created by applying other similarity search software to the same genome sequences. Storing large sets of MUMs also consumes more memory. (However, it has to be considered that the “real” memory usage is hard to determine due to the fact that the current implementation of GBDP is based on Java. Since the Java virtual machine uses a garbage collector, the actual amount of consumed memory depends on the selection of the maximum heap size, which depicts a trade-off between run-time and memory consumption. The test was performed using a maximum heap size of 8 GB to optimize run-time performance.)

### Algorithm performance on incomplete genomes

**Figure 7:**
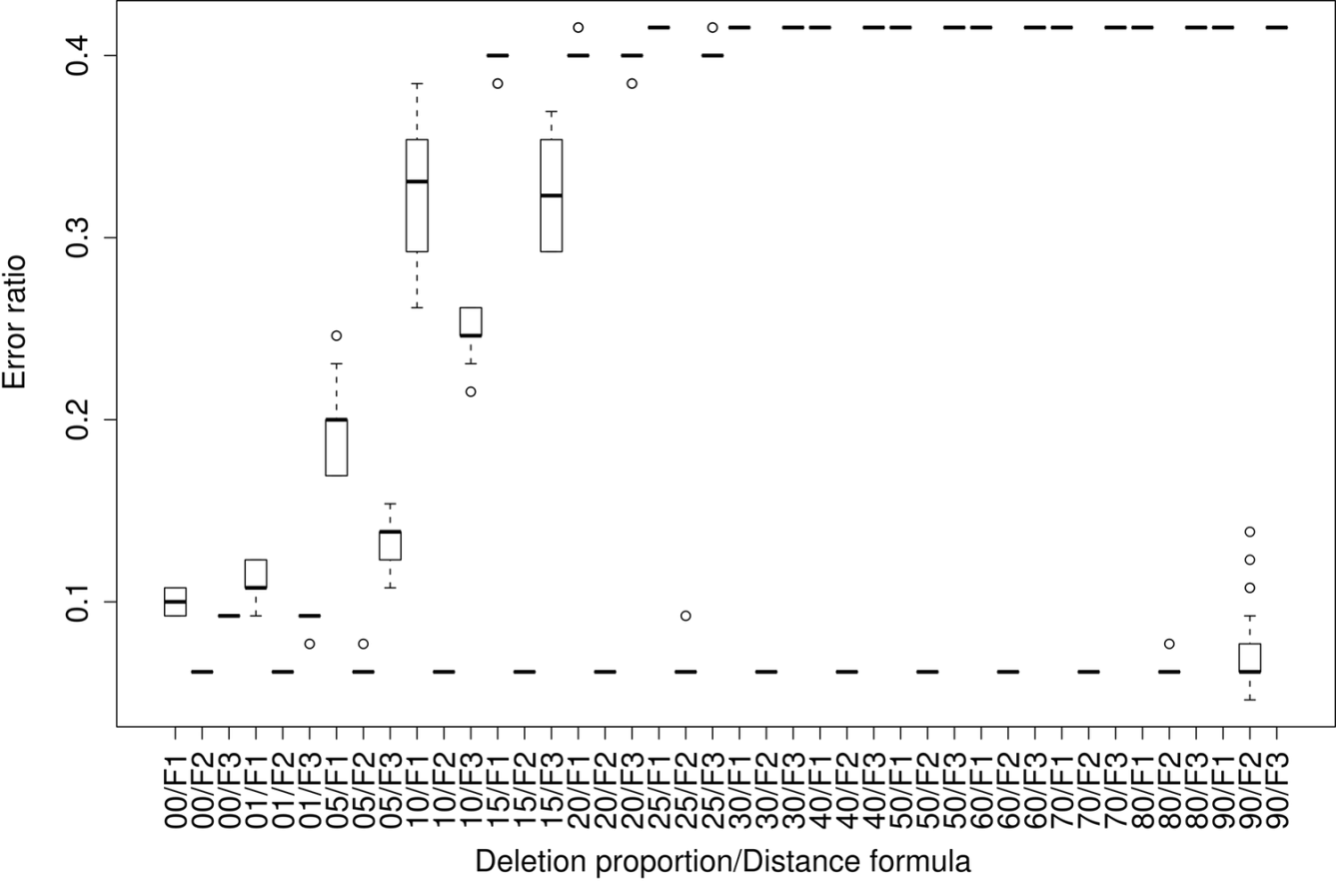
Boxplots showing the error ratios in predicting a DDH value ≤70% or >70% if applied to genomes artificially made incomplete. GGD were calculated using NCBI-BLASTN without filtering and all ten GBDP distance functions. The x-axis indicates the combination of the retained proportion of the genome (in percent) and the distance formula; F1, F2 and F3 refer to formulas (1), (2) and (3) as described above.

**Figure 8:**
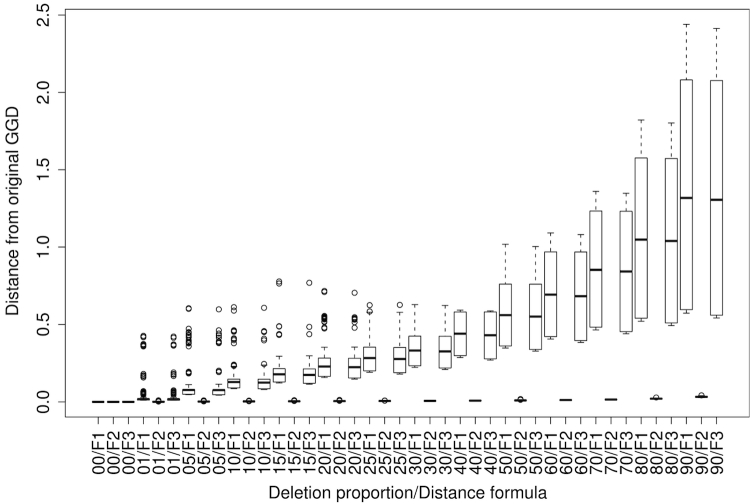
Boxplots showing the Euclidean distances between the original GGD inferred from complete genomes and those inferred from genomes artificially made incomplete. GGD were calculated using NCBI-BLASTN without filtering and all ten GBDP distance functions. The x-axis indicates the combination of the retained proportion of the genome (in percent) and the distance formula; F1, F2 and F3 refer to formulas (1), (2) and (3) as described above.

The error ratios in predicting a DDH value ≤70% or >70% if applied to genomes artificially made incomplete is shown in Figure 7 for NCBI-BLASTN without filtering and all ten GBDP distance functions. While the median error ratio over all functions and simulation replicates approaches more than 40% for 25% genome deletion, the error ratio of formula (2) and its logarithmic modification remains stable at 0.062 irrespective of the deleted genome proportion until 80% deletion (note that the simulation was based on a reduced dataset of 65 pairs of completely sequenced genomes). Similarly, Euclidean dissimilarities between the GGD derived from the original genomes and those inferred from the incomplete ones increase nearly linearly with increasing deletion proportion, but formula (2) remains visible at dissimilarities between 0.0005 and 0.0284 for 1% to 80% genome deletion (Figure 8).

## Discussion

### Best GGD algorithms for mimicking DDH

As expected, in our experiments, the error ratio of the prediction of DDH values smaller/larger than the 70% threshold by GGD had less discriminating power than calculating correlations between DDH and GGD. However, because those programs and settings that were optimal with Kendall's tau were also optimal regarding the error ratio, correlation can well be used to select the best programs and parameters. The situation is more complicated regarding the optimal GBDP distance function, which is not necessarily the same regarding correlation and error ratio. Therefore, a prediction of whether a DDH value would be at least as large as 70% can be based on the GGD thresholds resulting in the lowest error ratios.

Interestingly, the more sensitive programs or settings are not necessarily those with the highest correlation. For instance, MUMmer performs best with moderate minimum match lengths (Figure 3). Likewise, BLAT shows a higher correlation with the default tile size of 11 bp than with the supposedly more sensitive 8 bp. Moreover, WU-BLASTN, which usually results in larger sets of HSPs, including much shorter HSPs (personal observation), is outperformed by NCBI-BLASTN, and works better when filtering is applied (Figure 1). These results may be caused by the loss of information inherent to the DDH approach itself, which would explain why a corresponding loss of information caused by less sensitive settings for GGD calculation causes an increase in the correlation. The conclusion that DDH is imprecise is confirmed by the comparison with the 16S rRNA data – otherwise it would be hard to explain why GGD (and ANI) show a significantly higher correlation with 16S rRNA distances than does DDH. Accordingly, there may be inherent difficulties in obtaining a perfect correspondence between GGD and DDH because of the imprecision of the latter [8,9]. Thus, the high correlations and the minimal obtained error ratio of about 4% are already rather promising.

Running time and memory consumption of the distance calculation should not be overlooked as a selection criterion for GGD approaches. However, the best performing programs and/or settings, particularly NCBI-BLASTN and MUMmer, are also comparatively fast and need a feasible amount of RAM only (Table 1). The current GBDP implementation also seems to work well, even though in the current implementation all distance functions are exported at once, irrespective of whether they are processed further. A significant exception is both time and space consumption in the case of MUMmer combined with greedy-with-trimming, most likely because of the very large number of MUMs to cross-compare. However, because MUMmer has a built-in mechanism for avoiding non-unique matches, and, hence, a built-in ability to deal with repetitive sequences, greedy-with-trimming is unnecessary in that case and can safely be replaced by the much faster coverage distance (Figure 3, Figure 4; Table 1). Running time differences are less prominent for the HSP determining programs (Table 1); this, in addition to the likely benefit from greedy-with-trimming in the case of repetitive sequences [21,24], indicates that the latter algorithm should be used for BLASTN, BLAT and BLASTZ.

MUMmer also differs in another aspect from the HSP determining programs since distance formula (1) was optimal. However, this is hardly surprising because MUMs comprise exactly identical sequences only and, hence, the difference between formulas (1) and (3) might only be caused by minor numerical differences. In contrast, only formula (3) was optimal for the best-performing HSP-based approaches. This might have been expected because that formula preserves most information and also performed best in a phylogenetic context [35]. However, formula (2) achieved a lower error ratio at 70%, indicating that one and the same method-parameter combination does not necessarily perform equally well in mimicking DDH over the whole range of percent-wise similarities. Importantly, even though most researchers may only be interested in a GGD analog of the 70% threshold, overall high correlation cannot be dismissed because it indicates good replacement performance over the full range of possible thresholds. For instance, in overspeciated groups with comparatively little 16S rRNA divergence such as *Streptomyces*, establishing novel species is frequently accepted even in the case of higher DDH similarity [43].

In consequence, some uncertainty remains regarding an overall 'best' method for replacing DDH by the comparison of fully sequenced genomes. In our view, this problem is caused by the limited size of the empirical datasets currently available for testing, which, as mentioned above, is due to the low proportion of type strains among the organisms for which fully sequenced genomes are available [31,32]. Accordingly, only few 'hybridization groups' [17] can be tested, even though DDH and GGD results may depend to some degree on the phylogenetic position of such groups of closely related organisms. This highlights the necessity to (i) consider distinct GGD functions, as, e.g., included in the GBDP framework, and (ii) to provide an infrastructure that allows one to update the underlying empirical test datasets and the corresponding optimal parameters for calculating DDH analogs from GGD values [29].

Nevertheless, it is already noteworthy that the distance calculation with GBDP, although it has not been designed originally as a replacement for DDH, works as well or even outperforms the ANI and the 'Percent Conservation of DNA' approaches described earlier [17]. At the very least, this indicates that the first step applied in the latter algorithms, that is, to dissect the genomes into chunks of 1,000 bp, is obviously unnecessary. It seems to be more suitable to apply more generally useful approaches such as GBDP [21,24] to the challenge of surpassing the prediction power of DDH for species delineation by *in-silico* methods. The question remains, if such methods are also applicable to incompletely sequenced genomes.

### Use with incompletely sequenced genomes

Our simulation reveals that only one family of distance functions, i.e. formula (2) in either its original or logarithmized variant, is robust against the use of incomplete genomes; the error ratio of GGD based on formula (2) even remains constant throughout the investigated range of deleted proportions of the genome. This result is not unexpected though, as formula (2) is the only one independent of genome length, and thus it is not directly affected by the removal of HSPs due to the removal of parts of the genome. Although Henz *et al.* [24] observed that formula (1) performed best, their study was concerned with inferring deep phylogenetic relationships. The fact that formula (2) is more rapidly saturated with increasing 'true' evolutionary distance is hardly relevant for mimicking DDH by GGD because here the focus is on differentiating between closely related strains.

A shortcoming of our simulation approach is that the occurrence of read assembly artifacts cannot be taken into account. However, the incorrect arrangement of genome fragments is most likely not an issue for GBDP because the position of HSPs in the genome is not taken into account other than for determining overlapping HSPs and, hence, has no impact on the calculated distances. Furthermore, the trimming algorithm reduces the sets of HSPs to their non-overlapping subsequences and thus removes the effect of repetitive sequences [24], which are particularly often incorrectly assembled. A more important limitation of our simulation approach might be that it does not consider that sequence quality also decreases with decreasing coverage; the reads we have simulated are error-free by definition. Accordingly, constant error ratios down to 80% deletion (and perhaps more) are most likely an overestimation of GGD performance if applied to incomplete genomes. However, regarding that draft genome sequences (lacking gap closure) usually comprise 95-99% of the full genome length (in addition to the fact that gaps often comprise repeat regions, which are difficult to sequence and are removed by GBDP trimming anyway), our successful identification of distance functions that are robust against missing genome parts show great promise for future genome-based taxonomy.

## Conclusion

Our study confirms and extends the result of a previous study [17] that methods are available which outperform DNA-DNA hybridization similarities for microbiological species delineation on a broader data basis and for a broader range of methodologies. While several of the tested methods for HSP or MUM determination show high correlations and low error ratios, the apparently most widely used one, NCBI-BLASTN, performs at least as well as the others. Furthermore, we showed that distance functions devised for whole-genome phylogeny perform at least as well as methods described earlier, which are currently not publicly available. Additionally, we demonstrated that one family of distance functions is robust against the incompleteness of genome sequencing. Because the range of investigated methods was broader than in previous studies, we were able to provide an accompanying standard operating procedure for conducting HSP- or MUM-based genomic comparisons and a reference implementation available as a web service for genome-based species delineation [29].

Let alone the wealth of information of general interest available in complete genomes [28], replacing DDH by GGD is promising for taxonomists for several reasons. First and foremost, in contrast to DDH [3] it is possible to work incrementally, reusing sequenced genomes indefinitely. Also, in addition to DDH, determination of the G+C content *in vitro* and 16S rRNA sequencing of type strains can also be substituted by genome sequencing, which would result in more precise measures than any of the three approaches. By inferring pathways from annotated genome sequences it may even be possible to confirm, if not to infer, whether compounds that are routinely used in chemotaxonomy are produced by the strain under study [44,45]. The steadily decreasing sequencing costs will enable microbiologists to routinely use complete genomes irrespective of the question of which sequencing method(s) will become the most popular [28]. The present study confirms the view that methods for taxonomic analysis of genomic information are waiting in their wings and are not an obstacle for crossing the border into the era of genome-based taxonomy.

## Supplementary Material

Electronic supplementary information has been made available through the reading tools sidebar, containing the list of examined genomes, the correlations and error ratios for all methods and parameters and the underlying data (genome-to-genome distances) used to determine them.

## Notes

When preparing the revised manuscript an article by Richter and Rosello-Mora (PNAS 106:19126-19131) came to our attention, which also addresses the problem of how to replace DDH by approaches based on genome sequences. The latter authors provide a GUI-driven re-implementation of ANI but no web service. Only a single HSP-determining program is assessed, which is compared to an approach based on DNA strings (word counts). Regarding incompletely sequenced genomes, the results presented in the aforementioned publication are comparable to ours.
